# Bravery in the Ranks

**Published:** 1915-08-14

**Authors:** 


### Bravery in the Ranks.

### GALLANTRY OF THE R.A.M.C. ORDERLIES.

The Royal Army Medical Corps is again strongly represented in the list of over 400 non-commissioned officers and men who have been awarded the Distinguished Conduct Medal for acts of gallantry and devotion to duty "whilst serving with the Expeditionary Forces in France and Flanders, the Dardanelles, and Turkey in Asia."

Private W. R. Fitch, of the R.A.M.C., who is attached to the 1st Battalion of the Hampshire Regiment, was marching with a column of transports which was under very heavy shell fire at Ypres. The driver of a cart was shot, and the horses started to bolt, when he very courageously stopped the horses, thereby averting a congestion of traffic which under heavy shell fire would have led to great loss of life and stores. Of Corporal J. E. McNeill it is recorded that during the night of May 15-16, near Rue du Bois, he repeatedly went out and brought in wounded men under a heavy fire, showing great bravery and devotion to duty. At Festubert, when the stretcher-bearers of the advanced companies had all been killed or wounded, Private G. H. Mills, of the 2nd City of London Field Ambulance R.A.M.C. (T.F.), volunteered for this work and remained working under a heavy fire until every wounded man had been dressed. On previous occasions, it is added, Private Mills has shown the greatest devotion to duty. Sergeant J. Percy receives his medal for conspicuous gallantry throughout the campaign, especially at Festubert, from May 16 to 18, when he worked night and day under incessant fire bringing in the wounded. "Sergeant Percy," states the *London Gazette*, "has shown great resource and courage under heavy fire, and has set a fine example in devotion to duty."

Private W. Steedman, of the R.A.M.C. (attached 9th Lancers), displayed gallantry and ability as orderly to a medical officer at Ypres on May 13. The official report of his conduct is particularly striking. It says: "He was indefatigable in attending to the wounded. Under the heaviest fire he always went out to anyone reported wounded, and when the doctor was killed opposite the wide breach in the trench he at once went out and removed the body. Single-handed, he arranged later on for the removal of all the wounded during the night, and gave a splendid example of devotion to duty."

Sergeant H. W. Stoner, it is stated, has always shown conspicuous coolness and courage, and has given a fine example of devotion to duty. He is rewarded for great bravery and consistent good work throughout the whole campaign. He was frequently under very heavy fire, notably so on May 24, during the heavy fighting near Wieltje. Acting-Sergeant R. M. Watchorn, of the 3rd Welsh Field Ambulance R.A.M.C. (T.F.), was in the Wittepoort Farm, near Ypres, which was being used as divisional collecting post for the wounded. Though the farm, road, and neighbourhood were under heavy shell fire, he rode four miles on a motor-cycle to bring up dressings for the wounded, and on his return continued to assist in dressing them.

#### Australian Ambulance Men.

Two Australian Ambulance workers also figure in the list of new recipients of the Distinguished Conduct Medal.

Lance-Corporal V. Cawley, of the No. 2 Field Ambulance, 1st Australian Division, displayed conspicuous gallantry during the landing operations at the Dardanelles in the neighbourhood of Gaba Tepe. He advanced under heavy rifle and shrapnel fire, and spent the day in attending to wounded men. Repeatedly during the following days he brought in wounded men over ground swept by the enemy's fire.

Private M. D. Cowtan, of the 1st Australian Casualty Clearing Station, is reported to have been indefatigable during the first four days after the landing at Gaba Tepe, in giving aid and carrying water to the wounded, and his unswerving courage under fire was invaluable in its effect.

Note.—This article carries on the accounts, based on the official list of awards to the rank and file of the R.A.M.C., which appeared in The Hospital of July 10, p. 314. Each list of decorations will be similarly dealt with.

